# Experimental Study of Shenfu Injection on the Prevention and Treatment of Paclitaxel Chemotherapy DRG Neuron Injury

**DOI:** 10.1155/2020/8239650

**Published:** 2020-02-17

**Authors:** Panpan Lv, Xi Zhu, Jingyang Wang, Guangping Li, Xian Ying Shen, Jianlong Ke, Cui Wang, Shaoquan Xiong

**Affiliations:** Department of Medical Oncology, Hospital of Chengdu University of Traditional Chinese Medicine, Chengdu 610072, Sichuan, China

## Abstract

**Purpose:**

The purpose of this paper is investigating the effect and mechanism of Shenfu injection (a Traditional Chinese Medicine injection form) on prevention and treatment of paclitaxel chemotherapy in peripheral nerve injury.

**Methods:**

Wistar rat dorsal root ganglion cells were cultured in vitro and divided into groups of MOCK, PT, PT + LD, and PT + HD. Each group was cultured at a total serum concentration of 10%, including 10% blank serum in the MOCK group, 0.73 (IC30) *μ*mol/L paclitaxel + 10% blank serum in the PT group, and 10% and 5% drug-containing serum and equal amount of paclitaxel were added into the high- and low-dosage groups, respectively. After culturing for 24 hours, the following tests were performed: (1) cell proliferation detected by using CCK-8 and a microplate reader; (2) axon length detected by cellular immunostaining and detection analysis on antibody *β*-tubulin III; and (3) changes in mitochondrial membrane potential by analyzing immunofluorescence staining with JC-1 probe.

**Results:**

(1) Cell proliferation: OD values of the MOCK group and PT group were 0.43 ± 0.02 and 0.25 ± 0.03, respectively (*P* < 0.05), while OD values of groups PT + LD and PT + HD were 0.41 ± 0.05 and 0.46 ± 0.03, respectively, higher than group PT (*P* < 0.05), while OD values of groups PT + LD and PT + HD were 0.41 ± 0.05 and 0.46 ± 0.03, respectively, higher than group PT (*μ*mol/L paclitaxel + 10% blank serum in the PT group, and 10% and 5% drug-containing serum and equal amount of paclitaxel were added into the high- and low-dosage groups, respectively. After culturing for 24 hours, the following tests were performed: (1) cell proliferation detected by using CCK-8 and a microplate reader; (2) axon length detected by cellular immunostaining and detection analysis on antibody *μ*mol/L paclitaxel + 10% blank serum in the PT group, and 10% and 5% drug-containing serum and equal amount of paclitaxel were added into the high- and low-dosage groups, respectively. After culturing for 24 hours, the following tests were performed: (1) cell proliferation detected by using CCK-8 and a microplate reader; (2) axon length detected by cellular immunostaining and detection analysis on antibody *P* < 0.05), while OD values of groups PT + LD and PT + HD were 0.41 ± 0.05 and 0.46 ± 0.03, respectively, higher than group PT (*P* < 0.05), while OD values of groups PT + LD and PT + HD were 0.41 ± 0.05 and 0.46 ± 0.03, respectively, higher than group PT (

**Conclusion:**

Shenfu injection can prevent the toxicity of DRG neurons induced by paclitaxel, and its mechanism may be related to the alleviation of mitochondrial dysfunction.

## 1. Introduction

Chemotherapy has remained the mainstay of medical treatment in oncology, and its common side effects are not only gastrointestinal reaction, myelosuppression, liver and kidney injury but also chemotherapy-induced peripheral neuropathy (CIPN) [1]. It has been reported in the literature that the incidence of CIPN can be as high as 50%–80% [2]. The chemotherapy drugs that cause CIPN mainly include taxanes, platinum, and vincristine. There is still no specific method and drug for the prevention and treatment of this disease [3]. Based on our previous clinical observations, the use of Shenfu injection can improve the symptoms of CIPN [4]. Ultrastructural analysis under electron microscope in vivo experiments on rats found that Shenfu injection can also prevent the peripheral nerve damage caused by paclitaxel [5]. In this study, we cultured DRG neurons in vitro medium and firstly discussed the prevention and treatment of Shenfu injection on CIPN at the cellular level. The report is as follows.

## 2. Materials and Methods

### 2.1. Experimental Animals and Feeding Conditions

Forty, 250 ± 50 g, healthy, 3-week-old, male Wistar rats were provided by the Animal Experimental Center of Chengdu University of Traditional Chinese Medicine (batch number: SYXK (chuan) 2017-179) and were fed according to the standard conditions of experimental animals. This experimental program was reviewed by the Experimental Animal Ethics Committee.

### 2.2. Experimental Equipment and Reagents

Optical microscope (Japan OLYMPUS IX71), 36.5°C CO2 incubator (US THERMO FISHER), Shenfu injection (Yaan Sanjiu Pharmaceutical Co., Ltd., batch number 17110205003), paclitaxel injection (Haikou Pharmaceutical Co., Ltd., batch number 12180708), CCK- 8 (Japan DOJINDO), JC-1 probe (US THERMO FISHER), *β*-tubulin III, Alexa 488, DEME medium, DEME/F12 medium, PBS, collagenase type I, trypsin I, and Alexa 488 were purchased from Sigma.

### 2.3. Preparation of Drug-Containing Serum

Ten Wistar rats were injected at 8 ml/kg for 5 days. After 2 hours of the last injection, the rats were sacrificed by dislocation. The hearts were made to bleed and stand overnight at 4°C. The serum was separated by centrifugation, inactivated at 56°C for 30 min, and stored separately at −80°C (drug-containing serum). Another 10 rats were intraperitoneally injected with normal saline 8 ml/kg at the same time, and serum was taken after sacrifice (blank serum).

### 2.4. DRG Neuron Isolation and Culture

Referring to literature [6], the steps are briefly described as follows: The remaining 20 Wistar rats are decapitated and the medullary cavity is cut along the spine. The DRG was removed and digested with 1 mg/ml collagenase and 2.5 mg/ml trypsin. After centrifugation, PBS was stopped for recentrifugation, and DMEM medium (including dual antibody) was used for routine liquid exchange and subculture.

### 2.5. Paclitaxel IC30 Detection

The DRG cells were adjusted to 5 ∗ 107 cells/L and inoculated into 96-well plates at 100 *μ*l per well, and the cell fusion degree was 60–70%, after adding different concentrations of paclitaxel (2.0, 1.75, 1.5, 1.25, 1.0, 0.75, 0.1, 0.5, 0.25, 0.01, and 0 umol/L). The cells were cultured for 24 hours, and 10 *μ*l of CCK-8 solution (5 mg/ml) was added to each well for 30 min. The absorbance OD value of each well was measured by using a microplate reader to calculate paclitaxel IC30.

### 2.6. Group Administration

The DRG cells were cultured according to Section 2.4, and the DRG cells were passaged for 2-3 generations. Then, the cell culture medium containing DRG cells was transferred into 96-well plates (10000 cells/well) and 6 secondary wells in each group according to the following groups; then, they were given drug in the following groups:

  Mock: 10% blank serum
  PT: paclitaxel IC30 + 10% blank serum
  PT + LD: paclitaxel IC30 + 5% drug-containing serum + 5% blank serum
  PT + HD: paclitaxel IC30 + 10% drug-containing serum

### 2.7. Test Index

#### 2.7.1. Cell Proliferation

The above group was cultured for 24 hours and then detected by the CCK-8 method. The absorbance OD value of each well was measured at a wavelength of 620 nm by using a microplate reader.

#### 2.7.2. Axon Length

Referring to the literature [7] method, after coculturing for 24 hours, PBS rinsing, and 4% paraformaldehyde fixing, incubate primary antibodies *β*-tubulin III and secondary antibody Alexa 488 stain, and dark room fluorescence microscope observation was sequentially conducted, using Image J (v1.8.0) software analyzing the length values of axons in at least 100 neurons.

#### 2.7.3. Mitochondrial Membrane Potential Change

Referring to the literature [8] method, after 24 hours of group culture, follow the JC-1 reagent instructions, use fluorescence microscopy to analyze the fluorescence intensity representative of mitochondrial membrane potential, and record 1 × 10^4^ cells; the depolarization of the membrane potential Δ*ψ* was measured according to the intensity of red and green fluorescence.

### 2.8. Statistical Methods

The data were performed using spss20.0 software. The measurement data were expressed as mean ± standard deviation (*x* ± *S*). The *q*-test was used to compare the two groups. *P* < 0.05 was considered statistically significant.

## 3. Conclusion

### 3.1. Determination of Paclitaxel IC30

**Figure 1:**
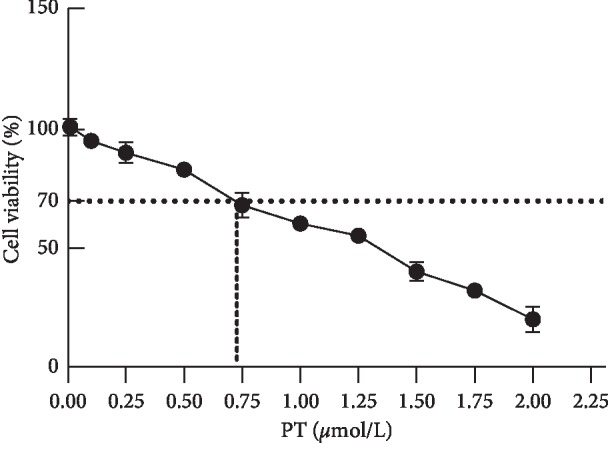
Paclitaxel antifungal susceptibility. The abscissa represents paclitaxel concentration, the ordinate represents the survival percentage of cell, and the dashed line corresponds to the paclitaxel concentration at IC30.

As shown in Figure 1, IC30 indicates 30% of the dead cells, that is, the concentration of paclitaxel at a mortality rate of 30%, and the IC30 of paclitaxel was calculated to be (0.73 ± 0.05) *μ*mol/L.

### 3.2. Cell Proliferation

**Figure 2:**
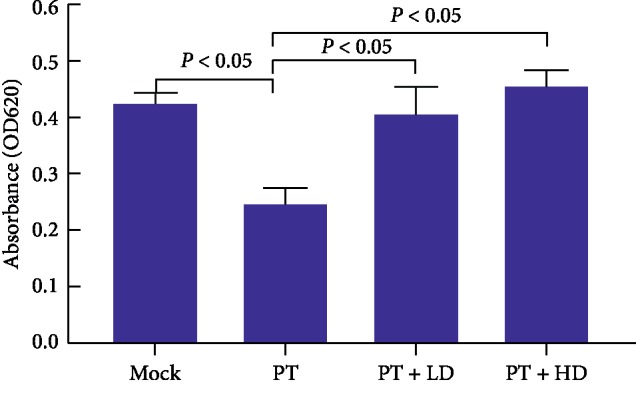
Proliferative capacity of cells in drug-containing serum. The vertical axis represents the absorbance OD value, and the absorbance OD value of each well was measured with a 620 nm wavelength microplate reader using CCK-8. The horizontal line in the figure represents a comparison between the two groups, *P* < 0.05.

After 24 hours of drug-adding culturing, the absorbance OD value represents the cell growth ability. The measured OD values are as follows: 0.43 ± 0.02 in the Mock group, 0.25 ± 0.03 in the PT group, 0.41 ± 0.05 in the PT + LD, and 0.46 ± 0.03 in the PT + HD group, and each group is compared as shown in Figure 2.

### 3.3. DRG Axon Length

**Figure 3:**
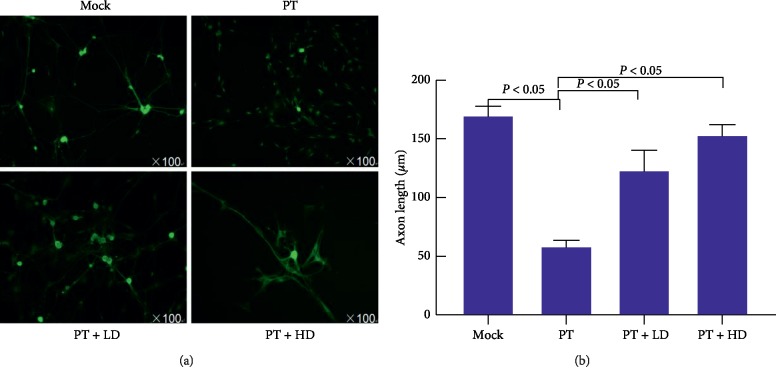
Growth of cells and axons in drug-containing serum. (a) Axon growth and color development. It was stained with *β*-tubulin III and pictures are zoomed in ×100 times darkroom. The difference in axon length between groups was obvious. (b) Quantitative analysis of axon length; Image J (v1.8.0) software analyzed the length of axons in each group. The horizontal line represents the comparison between the two groups, *P* < 0.05.

After 24 hours of drug-adding culturing, axonal staining can be performed by staining axons by staining cell axonal tubulin with *β*-tubulin III. Cell growth and color development are shown in Figure 3(a), and the length of axon is quantitatively analyzed as shown in Figure 3(b); the length of the axon was measured; the Mock group was (171.19 ± 9.2) *μ*m; the PT group was (59.2 ± 6.1) *μ*m; the PT + LD was (124.2 ± 18.3) *μ*m; and the PT + HD group was (154.5 ± 22.9) *μ*m.

### 3.4. Mitochondrial Membrane Potential Changes

**Figure 4:**
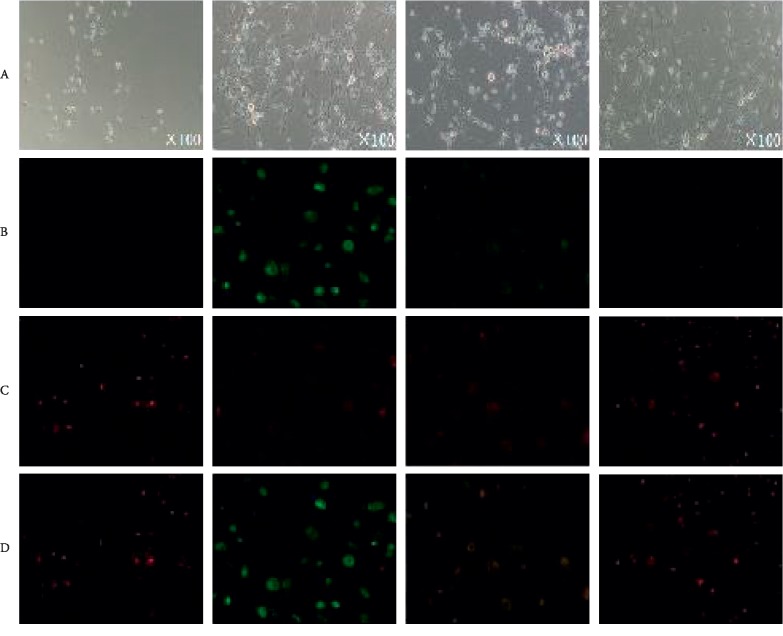
Shenfu injection inhibits mitochondrial membrane potential depolarization caused by chemotherapy. The observation under light microscope of (A) DRG cells was the white light photos without adding probe by taking pictures of ×100 times. B/C/D were obtained by different filters after the fluorescent staining of the daily probe. (B) JC-1 showed green fluorescence when the mitochondrial Δψm was lost, and the green fluorescence of the PT group was the most obvious. (C) The mitochondria Δψm showed red fluorescence when it was normal, and the red fluorescence of the MOCK and PT + HD groups was the most obvious. (D) B and C are shown in a fusion.

After incubation with JC-1 probes in each group, the cells were treated with red and green fluorescence, respectively, and the change of membrane potential Δ*ψ* was analyzed according to the fluorescence change. As shown in the figure, A is a white light picture, and B is coloring on green fluorescent, C is coloring on red fluorescent, and D is showing a fusion of B and C. Comparing with MOCK group, after DRG cells of PT group got treatment, the green fluorescence enhanced and red fluorescence weakened. Comparing PT + LD, PT + HD group and PT group, green fluorescence was weakened and red fluorescence was enhanced. See Figure 4 for details.

## 4. Discussion

Chemotherapy-induced peripheral neuropathy (CIPN), clinical manifestations of joint pain, numbness, and decrease of limb sensation extremity and so forth encounter cold aggravation; when it is severe, it affects the patient's quality of life, reduces treatment compliance, and even forces interrupting treatment [1, 3]. Chemotherapy drugs commonly used in clinical CIPN include taxanes (paclitaxel, docetaxel), platinum (oxaliplatin, cisplatin), vinblastine (vinblastine, vincristine), and so forth. Paclitaxel has been widely used in the treatment of lung cancer, esophageal cancer, head and neck tumors, breast cancer, gynecological tumors, and lymphoma in recent years. According to reports in the literature, the incidence of CIPN is as high as 59%–87% [9].

There is currently no specific drug treatment for CIPN, and ASCO does not currently recommend any preventive drugs [10]. However, for CIPN, which has occurred mainly with pain symptoms, ASCO recommends the use of duloxetine. It is also considered to give analgesic treatment to tricyclic antidepressants and gabapentin. However, the above drugs have no improving effect for cold pain and limbs numbness, and sensory disturbances [11], and there is no evidence that the above drugs have influence on the treatment of nerve repair.

Traditional Chinese Medicine has certain advantages in the prevention and treatment of CIPN. According to the symptoms, CIPN can be classified into the category of “arthritis” or “blood arthralgia” of Traditional Chinese Medicine [12]. Its main pathogenesis is the severe cold and pain feeling of chemotherapy, consuming yangqi, which causes the loss of primordial yang, lacking of warmth, lassitude of the extremities, and blood vessel obstruction, and if lasting for a long time, qi and blood will become weak, spoil channels, and have a feeling of extremities numbness, sensory disturbances, obstruction, and pain. Clinically used warming yang to benefit qi, activating blood to promote menstruation, and removing phlegm as well as relieving pain, and so forth have a good treatment effect on CIPN. Although the methods of nourishing blood, promoting blood circulation, and removing phlegm have certain treatment effect on CIPN, considering the damage of yang and arthritis of blood vessel is the basis of CIPN's syndrome, and the blood deficiency and the lack of blood vessel obstruction are secondary syndromes after the yangqi is damaged. From the simple to complicated perspective, this study focuses on the warming yang to benefit qi method, which includes the Shenfu injection, monkshood, and supplemental energy to help the yang, dispelling cold, and pain; ginseng, reinforce vital yuanqi, consolidate vessel, if using the two drugs together, both properties of the two drugs will come out. Clinically, it has been reported that Huangqi Guizhi Wu Wo Tang [13] reduces the incidence of oxaliplatin CIPN, and Tongmai Sini Decoction reduces the severity and incidence of CIPN [14]. However, clinical studies on paclitaxel have rarely been reported in the literature. In the previous study, our group adopted Shenfu injection in the treatment of CIPN and found that it can significantly reduce paclitaxel and oxaliplatin CIPN. Further research found that paclitaxel has more obvious effect on CIPN [4]. Further animal experiments found that Shenfu injection can improve the sciatic nerve injury of rats in ultramarine structure and reduce the destruction of endoplasmic reticulum as well as mitochondria in the subdevices of the suborganism [5]. Considering the clinical utility and improvement of organelles, this study is intended to, through the cell level, analyze the mechanism of prevention and treatment of paclitaxel CIPN by Shenfu injection.

Studies have shown [15] that paclitaxel can accumulate in peripheral nerves after administration, especially in DRG neurons, or it may cause damage first. Does Shenfu injection reduce DRG neuron damage and affect DRG axon growth? In this experiment, comparing with the blank group, paclitaxel alone can significantly inhibit DRG proliferation (*P* < 0.05), while the effect of cell growth inhibition after combining with Shenfu injection was significantly reduced (*P* < 0.05), and high-dose Shenfu injection effect is more obvious (see Figure 2). The use of DRG cells to study Traditional Chinese Medicine for CIPN is rarely reported.

The good growth of DRG represents as well cell morphology, intact capsule, and normal extension of axons, playing the role of transporting nutrients and metabolites and transmitting nerve impulses [16]. In this experiment, we also analyzed the axon growth of each group to evaluate the drug efficacy. Axon structural protein microtubules and axons with immunofluorescence staining were performed to observe cell growth and axon growth length more vividly under fluorescence microscope, as shown by the results: under the microscope, the paclitaxel group showed crisped at the cell edge of the blank group, the axon was shortened and thinned, and the cell edge was crisped, but after the combination of Traditional Chinese Medicine, it can be seen that the cell morphology was relatively good, and the cell morphology and axon growth were close to the normal control group. Quantitative analysis of the length of axons in each group showed that the length of axon cells in the paclitaxel group was significantly shorter than that in the blank group (*P* < 0.05). After combining with Shenfu injection, the axon length was significantly restored (*P* < 0.05), and the high-dose traditional Chinese medicine had a better effect on axon growth. The above suggested that Shenfu injection can improve the cell damage caused by paclitaxel, especially in terms of morphological recovery and axon growth. This is similar to the improved morphology of sciatic nerve cells and the improvement of axonal growth observed in our previous study under the electron microscope [5].

Among the life activities that influence neurons, the cellular subdevice-mitochondria plays an important role. After mitochondria damaging, transmembrane potential (Δ*ψ*) will undergo loss, respiratory chain electron transport disorder, oxidative phosphorylation decoupling, cellular ATP synthesis disorder, generate a large number of reactive oxygen species (ROS), activate mitochondrial apoptosis signals, and lead to nerves Meta-axial mutagenesis, which has paresthesia in extremity. In the previous study, our group used Shenfu injection to reduce the sciatic nerve degeneration caused by chemotherapy in rats. Electron microscopy showed that the sciatic nerve cell morphology was good, the axonal degeneration was reduced, and mitochondrial swelling was alleviated [5], suggesting that Shenfu injection has a curative effect on CIPN. In this experiment, we performed the following preliminary analysis on the effects of Shenfu injection on mitochondrial function at the cellular level.

JC-1 is generally used for the detection of mitochondrial membrane potential changes in early apoptosis [8]. For normal cells, JC-1 polymerizes in the mitochondrial matrix and shows red fluorescence, when the mitochondrial membrane potential is lost, JC-1 exists as a monomer in the cytoplasm showing green fluorescence, and the depolarization of Δ*ψ* is measured according to the intensity of red/green fluorescence. As shown in Figure 4, compared with the blank group, paclitaxel chemotherapy can increase the green fluorescence in DRG cells, while the red color is weakened, indicating that the membrane potential Δ*ψ* is depolarized, and the red fluorescence, that is, the membrane potential Δ*ψ*, is partially restored after being combined with the Shenfu injection. This suggests that Shenfu injection can restore mitochondrial membrane potential and maintain mitochondrial function. The above results indicate that paclitaxel-induced peripheral neurotoxicity may be related to the destruction of mitochondrial membrane integrity and mitochondrial damage, which is the first step of Traditional Chinese Medicine to regulate mitochondrial function and CIPN improvement study.

At present, the Traditional Chinese Medicine of warming yang and benefiting qi, promoting blood circulation and removing blood stasis (mainly Huangqi Guizhi Wuwu Tang) is used to treat the rat serum NGF, TRPs protein expression, ROS detection, NR2 mRNA expression, pNF-H upregulation, and so forth [17, 18], and only for oxaliplatin, it has not been tested for paclitaxel mechanism. Although the specific mechanism of CIPN is still unclear, our research has certain guiding significance for clinical practice. It is also the first time that Traditional Chinese Medicine has been researched on paclitaxel CIPN mechanism in home and abroad, which has important clinical and theoretical value and deserves further analysis. It should be pointed out that this experiment is a preliminary study. It has to be pointed out that the effect of Traditional Chinese Medicine on mitochondria is still not deep enough, and it still needs to be improved, such that if there is flow cytometry, the results of additional test of membrane potential will be more obvious.

## Data Availability

The data used to support the findings of this study are available from the corresponding author upon request.
